# 

**Published:** 1919-02-15

**Authors:** 


### Medical Appointments and Promotions.

### FOR SERVICES IN EAST AFRICA.

Among the appointments and promotions made for services rendered in connection with the operations in East Africa are the following:

*C.M.G.*—Temp. Lt.-Col. Hugh Basil Greaves Newham, M.D., M.R.C.S., R.A.M.C.; Maj. (A./Lt.-Col.) Richard Edmond Humphrey, R.A.M.C.

*C.I.E.*—Lt.-Col. (temp. Col.) William Wellesley Clemesha, Indian Medical Service.

*O.B.E.* (*Military Division*).—Capt. C. P. B. Wall, M.P., South African Medical Corps; Temp. Capt. A. G. Eldred, Spec. List (Nyasaland Medical Service); Temp. Capt. C. R. Howard, R.A.M.C.; Temp. Capt. Q. Madge, R.A.M.C.; Temp. Capt. L. R. H. P. Marshall, R.A.M.C.; Temp. Capt. S. Mason, S. African Medical Corps; Capt. G. McG. Millar, M.B., Indian Medical Service; Temp. Capt. A. J. M. Saville-Farr, R.A.M.C.; Temp. Major R. Semple, M.D., R.A.M.C.; Temp. Major E. Standish-White, F.R.C.S.I., R.A.M.C., attd. Northern Rhodesia Medical Corps.

*To be Brevet Major.*—Capt. (A./Lt.-Col.) J. D. Kidd, M.C., M.B., R.A.M.C.; Capt. (A./Lt.-Col.) J. A. Manifold, D.S.O., M.B., R.A.M.C.; Capt. E. A. Sutton, M.C., R.A.M.C.

*Military Cross.*—Capt. A. Robertson, M.B., R.A.M.C.

